# Spatial Patterns of Sea Level Variability Associated with Natural Internal Climate Modes

**DOI:** 10.1007/s10712-016-9386-y

**Published:** 2016-10-04

**Authors:** Weiqing Han, Gerald A. Meehl, Detlef Stammer, Aixue Hu, Benjamin Hamlington, Jessica Kenigson, Hindumathi Palanisamy, Philip Thompson

**Affiliations:** 1 Department of Atmospheric and Oceanic Sciences, University of Colorado, UCB 311, Boulder, CO 80309 USA; 2 Climate and Global Division, National Center for Atmospheric Research, Boulder, CO 80307 USA; 3 Oceanography, Remote Sensing of the Earth System and Coupled Climate Assimilation, Institut für Meereskunde, Universität Hamburg, Bundesstr. 53, 20146 Hamburg, Germany; 4 Department of Ocean, Earth and Atmospheric Science, Old Dominion University, Norfolk, VA 23529 USA; 5 LEGOS – OMP – CNES, UMR5566, 31401 Toulouse, CEDEX 9, France; 6 University of Hawaii Sea Level Center, Honolulu, HI 96822 USA

**Keywords:** Spatial patterns of sea level, Climate modes, Decadal sea level variability, Regional sea level change

## Abstract

Sea level rise (SLR) can exert significant stress on highly populated coastal societies and low-lying island countries around the world. Because of this, there is huge societal demand for improved decadal predictions and future projections of SLR, particularly on a local scale along coastlines. Regionally, sea level variations can deviate considerably from the global mean due to various geophysical processes. These include changes of ocean circulations, which partially can be attributed to natural, internal modes of variability in the complex Earth’s climate system. Anthropogenic influence may also contribute to regional sea level variations. Separating the effects of natural climate modes and anthropogenic forcing, however, remains a challenge and requires identification of the imprint of specific climate modes in observed sea level change patterns. In this paper, we review our current state of knowledge about spatial patterns of sea level variability associated with natural climate modes on interannual-to-multidecadal timescales, with particular focus on decadal-to-multidecadal variability. Relevant climate modes and our current state of understanding their associated sea level patterns and driving mechanisms are elaborated separately for the Pacific, the Indian, the Atlantic, and the Arctic and Southern Oceans. We also discuss the issues, challenges and future outlooks for understanding the regional sea level patterns associated with climate modes. Effects of these internal modes have to be taken into account in order to achieve more reliable near-term predictions and future projections of regional SLR.

## Introduction

Sea level rise (SLR) is an important indicator for climate change, with direct impacts on coastal society and island countries and far-reaching effects on global population and economy. For this reason, there is huge societal demand for improved projections of future sea level change, particularly at local scale along coastlines (e.g., Milne et al. 2009; Church et al. 2011, 2013; National Research Council (NRC) Report 2012). In situ and satellite observations show that during the past few decades regional changes of sea level can deviate considerably from the global mean. For instance, since the early 1990s the rate of SLR trend in the western tropical Pacific was about three times the global mean value, whereas in the eastern tropical Pacific sea level varied very little (e.g., Merrifield 2011; McGregor et al. 2012; Han et al. 2014a; Thompson et al. 2014). These regional changes are shown to be associated with basin-wide spatial patterns, which exhibit distinct decadal variations (e.g., Lee and McPhaden 2008). For simplicity, in this paper “decadal variability” is collectively referred to as variations from one to a few decades (including multidecadal trend).

Various factors can cause sea level to change at regional or local scales (e.g., Stammer et al. 2013): changes in atmospheric and oceanic circulations (often referred to as dynamic change), large-scale deformation of ocean basins, variation in Earth’s gravity field and local land movement (e.g., Church et al. 2013; Stammer et al. 2013; Kopp et al. 2015). Dynamic sea level change induced by changes in atmospheric and oceanic circulations is a major cause for contemporary decadal sea level variability as opposed to long-term anthropogenic changes, and a large fraction of the dynamic sea level change can be associated with natural internal climate modes in the Earth’s coupled climate system (Stammer et al. 2013).

In this review, we summarize our current state of knowledge regarding the spatial patterns of sea level variability associated with natural climate modes, with particular emphasis on decadal timescales. We will also identify major science issues and challenges for understanding and extracting the imprints of internal climate modes in observed sea level change patterns, with a hope of contributing to decadal sea level predictions, which emerge as pressing priorities in climate research today (e.g., Goddard et al. 2009; Hurrell et al. 2009; Meehl et al. 2009, 2014; Pohlmann et al. 2009; Doblas-Reyes et al. 2011; Polkova et al. 2015). In Sects. 2–5 below, we review our current understanding of spatial patterns of sea level variability associated with climate modes in the Pacific, Indian, Atlantic, and Arctic and Southern Oceans, respectively, delineating the related oceanic processes whenever possible. In Sect. 6, we first provide a summary and then discuss remaining issues and challenges for future research on sea level variability associated with climate modes.

## The Pacific

### PDO-Related Sea Level Patterns

In the western tropical Pacific Ocean, intensified SLR has been observed since the early 1990s compared to the preceding decades (e.g., Merrifield 2011). Modeling studies suggest that warming of the tropical Indian and Atlantic Oceans enhances surface easterly trade winds and thus contributes to the intensified SLR (e.g., Luo et al. 2012; Han et al. 2014a; Hamlington et al. 2014; England et al. 2014; McGregor et al. 2014); however, a large portion of this rapid SLR—together with weak falls in the eastern basin—is part of the basin-scale sea level pattern associated with the Pacific Decadal Oscillation (PDO) or decadal variability of ENSO (Bromirski et al. 2011; Merrifield et al. 2012; Meyssignac et al. 2012; Zhang and Church 2012; Hamlington et al. 2013, 2014; Moon et al. 2013; Han et al. 2014a; Thompson et al. 2014; Palanisamy et al. 2015). The PDO is defined as the leading empirical orthogonal function (EOF) of sea surface temperature (SST) anomaly over the North Pacific (>20°N), and the leading principal component (PC1) is referred to as the PDO index (e.g., Mantua et al. 1997; Minobe 1997; Zhang et al. 1997; Garreaud and Battisti 1999; see review papers by Alexander 2010; Liu 2012). It is significantly correlated with ENSO (e.g., Alexander et al. 2002; Newman et al. 2003; Deser et al. 2004; Schneider and Cornuelle 2005; Vimont 2005). On decadal timescales, the PDO is highly correlated with ENSO (Zhang and Church 2012) and the Interdecadal Pacific Oscillation (IPO), a basin-wide decadal climate mode associated with decadal SST variability in the Pacific (e.g., Power et al. 1999; Folland et al. 2002; Meehl and Hu 2006). The correlation coefficients for PDO-IPO and IPO-NINO3.4 indices (8-year low-passed) are both 0.88 over the period of 1900–2008 (Han et al. 2014a; Zhang and Church 2012). Given their high correlations on decadal timescales, it has been suggested that the IPO may not be confidently treated as an independent climate mode to ENSO decadal variability (e.g., Trenberth et al. 2007) or that the PDO is a statistic mode rather than a physical mode with a single mechanism (see review by Newman et al. 2016). For consistency, unless specified otherwise, we will use the term PDO hereafter to represent decadal ENSO variability, PDO and IPO.

**Fig. 1:**
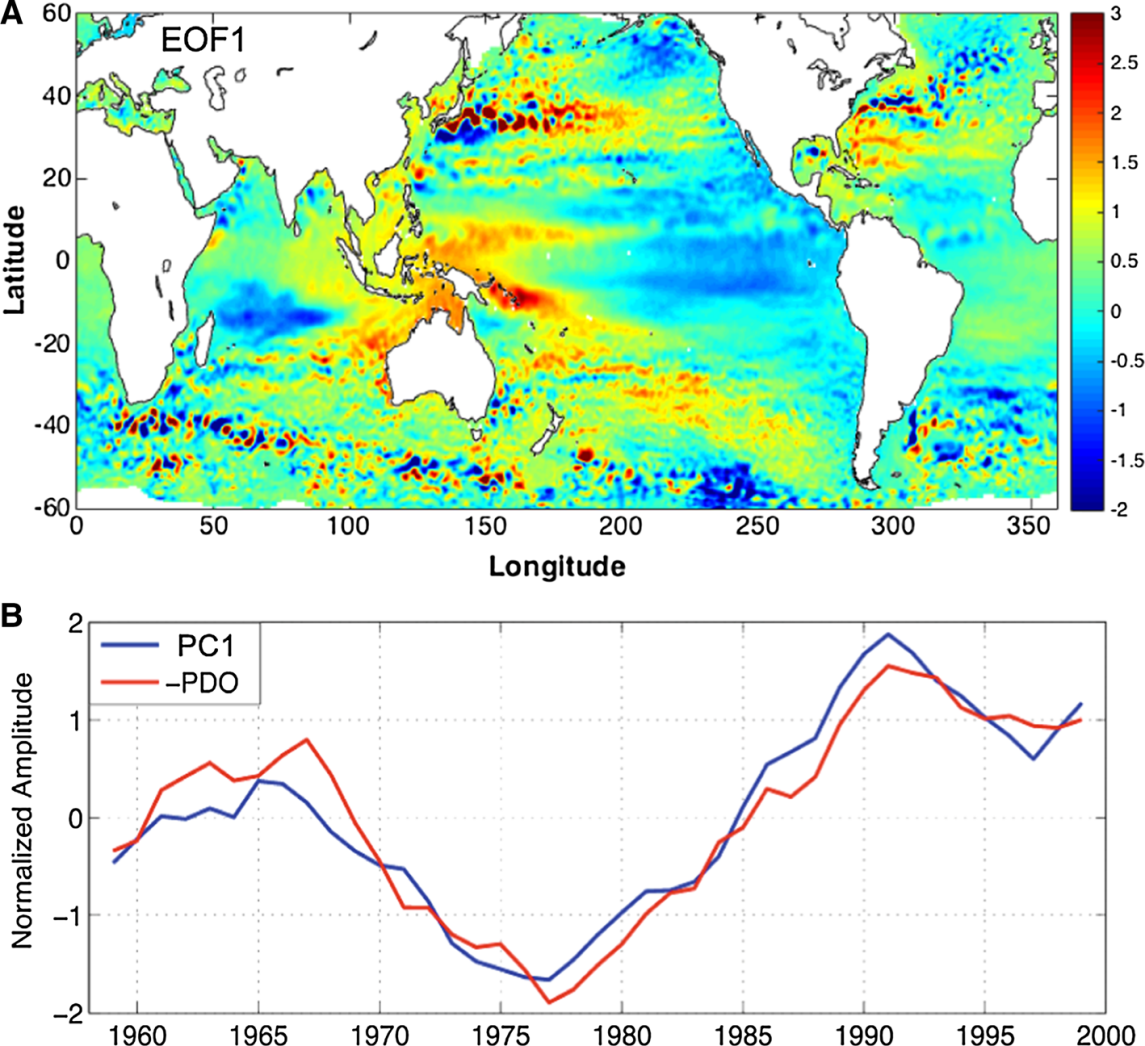
**a** Spatial pattern of the leading EOF (EOF1) of 20-year sliding trends of reconstructed sea level dataset (Hamlington et al. 2011) for the 1958–1999 period, **b** principal component (PC) for the leading EOF (*blue*) and reversed PDO index (*red*). Customized from Hamlington et al. (2013)

**Fig. 2:**
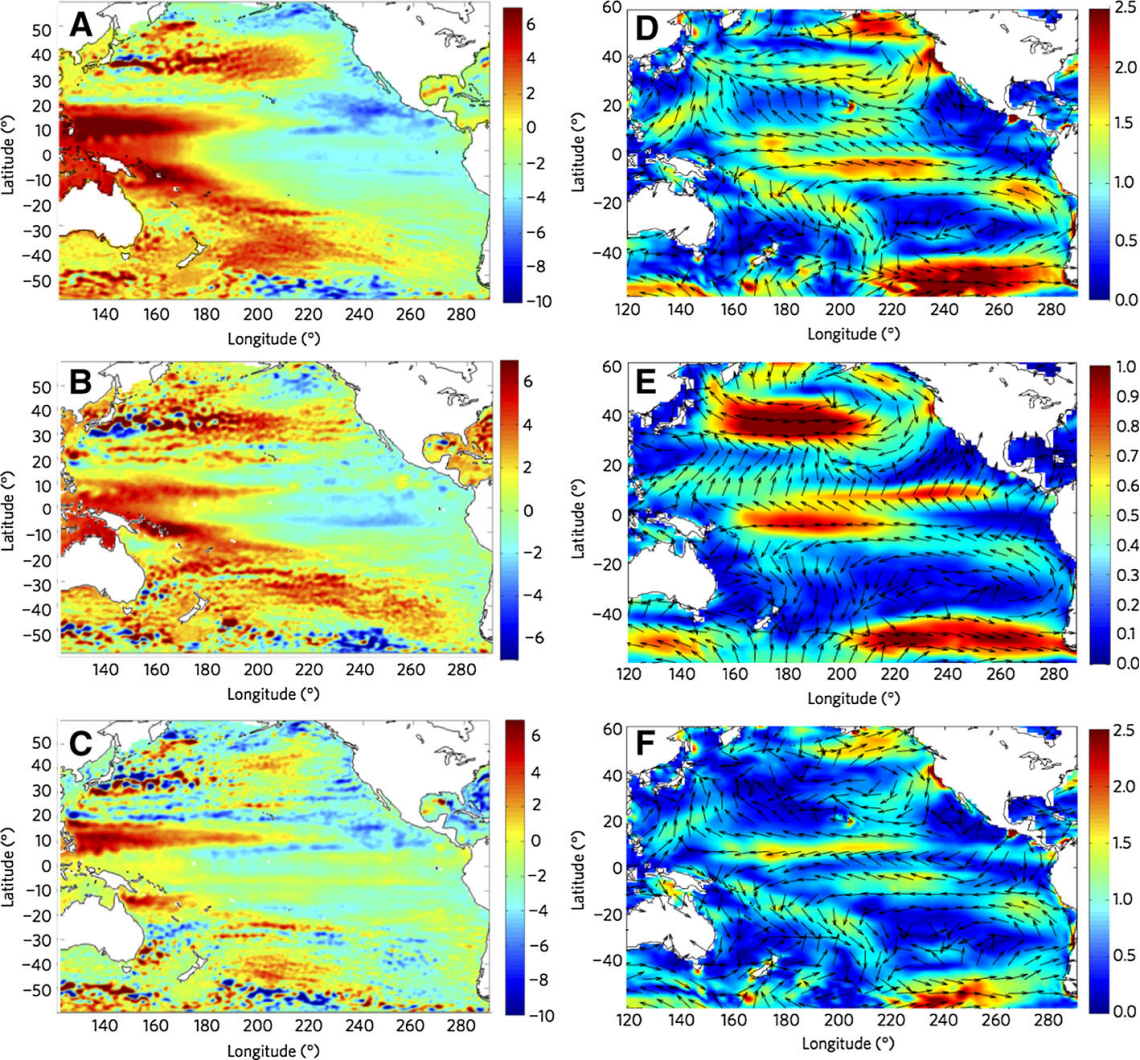
**a** Satellite-observed sea level trends (mm year^−1^) from 1993 to 2010 for Archiving, Validation and Interpretation of Satellite Oceanographic (AVISO) data, **b** PDO contribution estimated by the leading EOF of reconstructed sea level (see Fig. 1), **c** AVISO minus the PDO contribution, which is panel **a** minus panel **b**. The global mean sea level trend has been removed from the AVISO data. **d** Wind stress trends (mPa year^−1^) from 1993 to 2010 from Operational Ocean Re-Analysis Series 3 (ORA-S3) data; **e** PDO contribution, **f** ORA-S3 trends minus PDO contributed trends, which is panel **d** minus panel **e**. Customized from Hamlington et al. (2014)

**Fig. 3:**
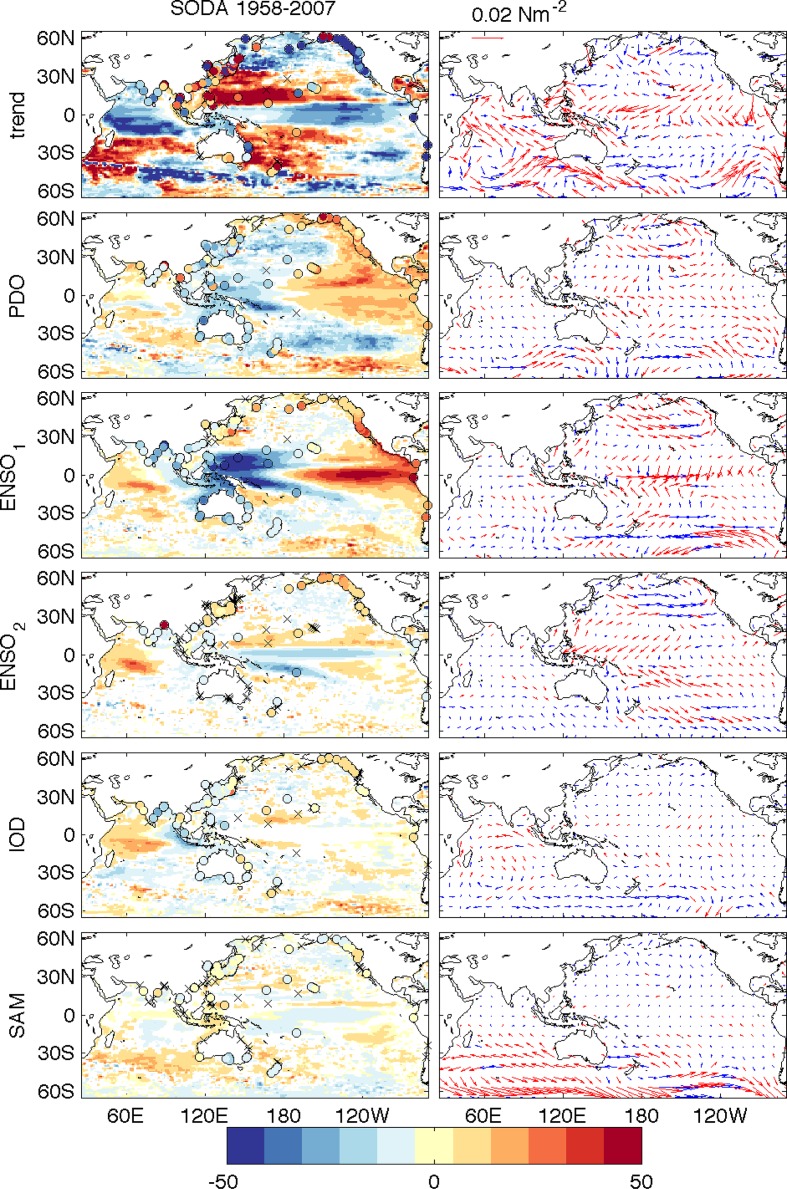
(*Left column*) Regression coefficients between climate indices and sea level from 50-year (1958–2007) SODA product over 65°S–65°N of the Indo-Pacific basin (25°E–70°W), (*Right column*) Regression coefficients between climate indices and surface wind stress from ERA40 wind before 2001 and ERA-Interim wind after 2001. Tide gauges are shown as *colored circles*, and *crosses* indicate where the tide gauge regressions are not significant. *Red vectors* indicate significance at 95 % level. All indices were smoothed using a 5-month running mean with long-term mean seasonal cycle removed. The PDO index has the high-frequency component removed by smoothing the monthly PDO index with successive 25- and 37-month running mean. Using IPO index yields similar results. The ENSO indices represent interannual variability, having the low-frequency component removed. The first ENSO index (ENSO1) is the commonly used Multivariate ENSO Index, and ENSO2 describes the nonlinear atmospheric response to SST anomalies associated with the combination of ENSO and the annual cycle (see Frankcombe et al. 2015 for details). From Frankcombe et al. (2015)

Both EOF analysis and multiple linear regression have been used to obtain the basin-scale spatial patterns of sea level variations associated with the PDO. Hamlington et al. (2013) performed EOF analysis on 20-year sliding trend maps of the annual mean reconstructed sea level data (e.g., Hamlington et al. 2011) for the 1950–2010 period, and showed that the leading EOF (EOF1) of global sea level change exhibits distinct spatial patterns (Fig. 1a), with SLR in the western tropical Pacific and Subtropical Gyre regions corresponding to sea level fall in the eastern basin in both hemispheres during a negative phase. Its temporal variability (PC1) is highly correlated with the PDO index, with a correlation coefficient of 0.96 (Fig. 1b) (see also Di Lorenzo et al. 2008). Consequently, EOF1 was defined as the PDO-related sea level variability, which explains 41 % variance (Hamlington et al. 2013) and dominates the satellite-observed, basin-wide sea level trends from 1993 to 2010 (Hamlington et al. 2014; Fig. 2a–c). The EOF1 patterns shown in Fig. 1a resemble the multiple linear regression patterns over the Pacific Ocean (e.g., Zhang and Church 2012; Si and Xu 2014; Frankcombe et al. 2015) (second row of Fig. 3) by regressing the observed, ocean model-simulated and reanalysis sea level data onto the PDO index. Regionally, they are similar to the North Pacific EOF1 of the upper 500 m thermosteric sea level from 1950 to 1998 (Lombard et al. 2005) and tropical Pacific EOF1 of reconstructed sea level from 1950 to 2009 (Meyssignac et al. 2012). Many studies have shown that decadal sea level variability in different regions of the Pacific including the marginal seas and US west coast is significantly correlated with the PDO index (e.g., Bromirski et al. 2011; Merrifield 2011; Marcos et al. 2012; Zhang and Church 2012).

**Fig. 4:**
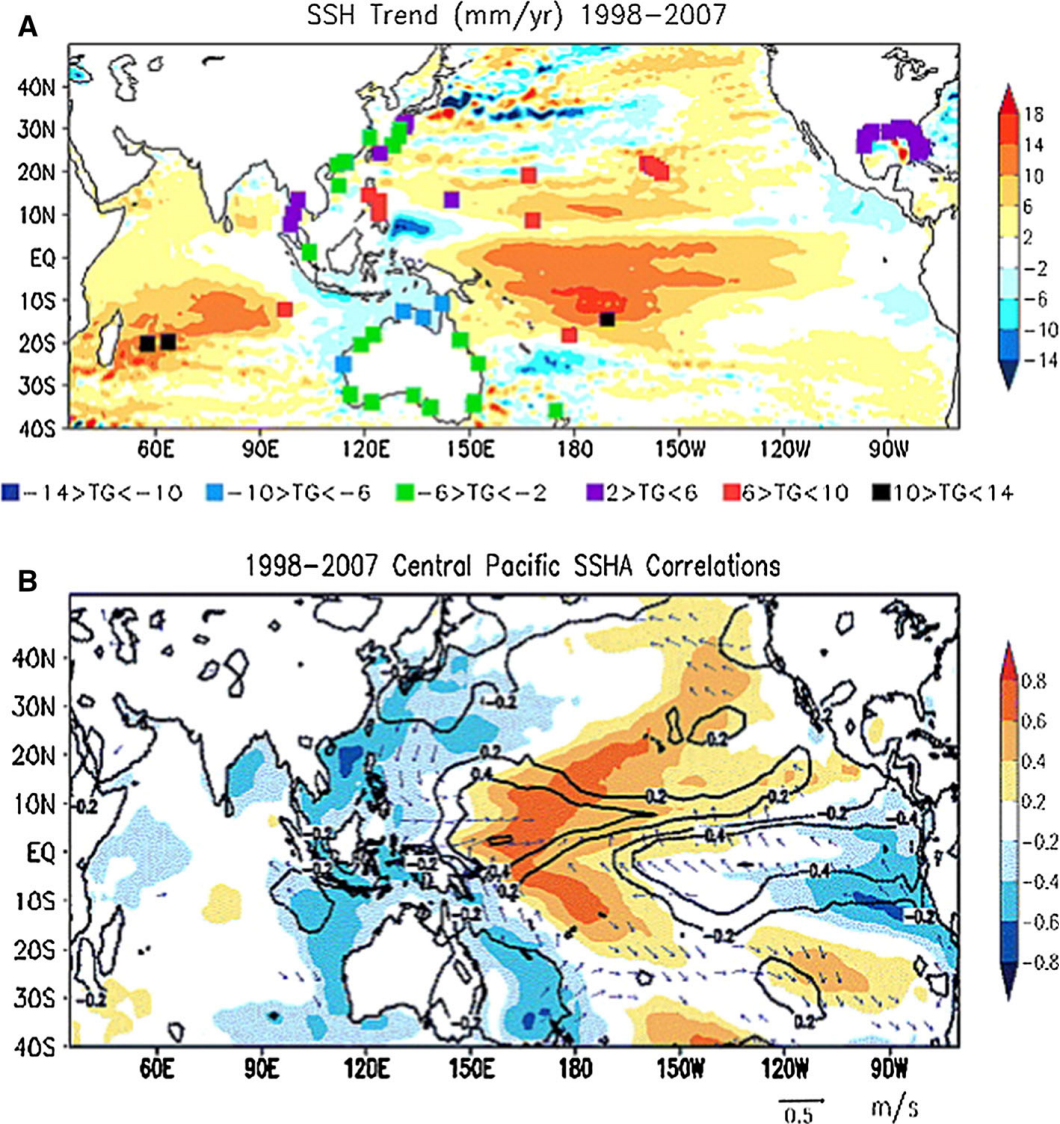
**a** The Indo-Pacific sea level trends for the period of 1998–2007. The *squares* denote the trends in sea level anomalies derived from the tide gauge satiations. **b** The central Pacific sea level correlation with anomalies of SST (*shaded*) and rainfall (*contour*) and its regression with surface wind anomalies for the decade of 1998–2007. Values below the 95 % confidence level based on a 2-tailed *t* test are not shown. From Behera and Yamagata (2010)

For the period of 1998–2007, the decadal trend of sea level shows evidently different spatial patterns: Sea level is higher than normal in the central Pacific flanked by lower than normal sea level on either side of the basin (Fig. 4a). Compared to the PDO-related sea level patterns since the 1950s (Figs. 1, 3), this abnormal condition is due to the frequent occurrence of El Niño Modoki (or central Pacific El Niño) events during 2000–2004, which are associated with wind convergence to the dateline (Behera and Yamagata 2010; Fig. 4b). The sea level rise in the central Pacific succeeded a phase of lower than normal sea level associated with La Niña Modoki events toward the end of the 1990s (Behera and Yamagata 2010). This result suggests that decadal changes in ENSO behavior and its associated winds will induce changes in the spatial patterns of decadal sea level variations.

### NPGO-Related Sea Level Patterns

**Fig. 5:**
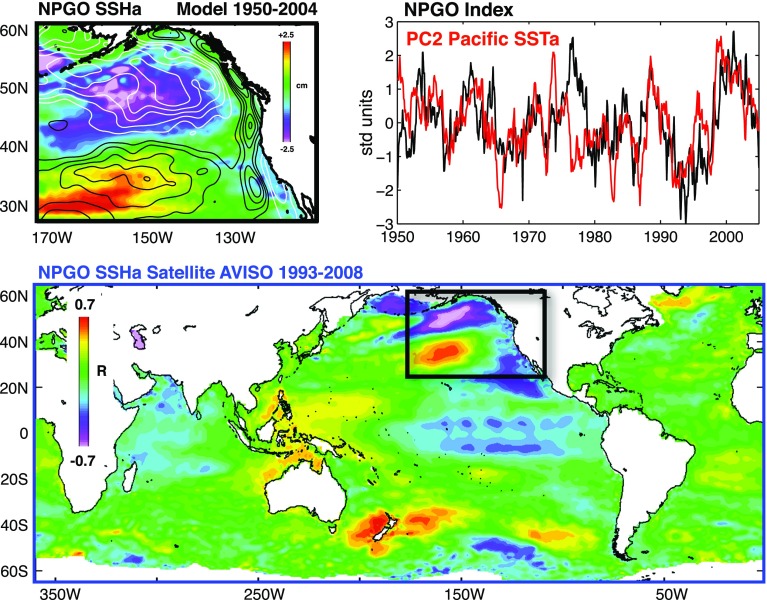
(*Top left*) The 2nd EOF pattern of monthly SSH anomalies from the output of a regional ocean general circulation model (OGCM) for the 1950–2004 period; (*top right*) The NPGO index (*black curve*) defined as PC2 of SSH anomalies (SSHa) from the OGCM output, and PC2 of Pacific SST anomalies from NOAA ERSST v3 from 1950 to 2004 (*red curve*); (*Bottom*) Correlation map between NPGO index and monthly mean AVISO SSHa or the 1993–2008 period. Figure provided by Dr. Emanuele Di Lorenzo

In addition to the PDO, the North Pacific Gyre Oscillation (NPGO) is also associated with distinct spatial patterns of sea level change. The NPGO is defined as the second EOF of sea surface height anomaly (SSHa) over the Northeast Pacific region (180°W–110°W; 25°N–62°N) (Di Lorenzo et al. 2008). Its spatial patterns reflect a pair of counter-rotating gyres, with a positive NPGO corresponding to low sea level of the Alaskan Gyre in the north and high sea level in the Subtropical Gyre to the south (Fig. 5). It is argued that the NPGO is not limited to the Northeast Pacific, but exhibits global signatures in both SSH (Fig. 5, bottom panel) and SST fields (Di Lorenzo et al. 2008). Indeed, Merrifield (2011) showed that decadal sea level variations in some regions of the western tropical Pacific have higher correlations with the NPGO than with the PDO.

### Forcing and Processes

**Fig. 6:**
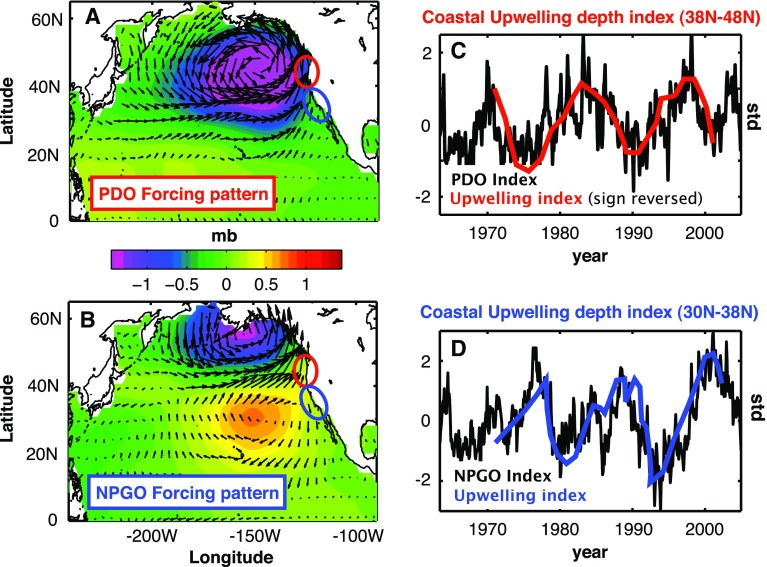
Regression maps of **a** PDO and **b** NPGO indices with NCEP wind stress vectors and sea level pressure (*color scale*), **c** Coastal upwelling depth index from inverse model calculations averaged from 38°N to 48°N (area denoted by *red circle* in Fig. 6a, b) compared to PDO index, **d** Coastal upwelling depth index averaged from 30°N to 38°N (area denoted by *blue circle* in Fig. 6a, b) compared to NPGO index. A positive upwelling index implies a deeper upwelling cell. Adapted from Di Lorenzo et al. (2008)

Previous studies suggest that over the past few decades change in wind forcing is the main cause for decadal sea level variability in the Pacific, including the intensified SLR in the western tropical Pacific since the early 1990s (e.g., Qiu 2002; Carton et al. 2005; Bindoff et al. 2007; Köhl et al. 2007; Köhl and Stammer 2008; Roemmich et al. 2007; Timmermann et al. 2010; Merrifield and Maltrud 2011; McGregor et al. 2012; Nidheesh et al. 2013; Qiu and Chen 2012; Han et al. 2014a). Given that a significant portion of the basin-scale coherent sea level pattern is associated with the PDO and NPGO, one can conclude that it is the surface wind changes associated with the PDO and NPGO that are the major cause for the basin-wide sea level patterns. Along the same line of arguments, regression analyses indeed show that PDO- and NPGO-related wind stress curl and alongshore wind (Fig. 6) can cause decadal sea level variations in the ocean interior and along the coasts by inducing Rossby waves (e.g., Qiu 2002), coastal Kelvin waves (e.g., Clarke and Lebedev 1997; Thompson et al. 2014), upwelling, and horizontal advection (Di Lorenzo et al. 2008; Bromirski et al. 2011). Hamlington et al. (2014) extracted the basin-wide surface winds associated with the PDO by regressing surface winds available from the European Centre for Medium-Range Weather Forecasts (ECMWF) operational ocean analysis/reanalysis system (ORA-S3) (Balmaseda et al. 2008) onto sea level PC1 from 1950 to 2010. The authors found that the linear trend of PDO-related wind from 1993 to 2010 (Fig. 2e) can explain a large portion of the observed trend (Fig. 2d). Note that while the PDO-winds have a single gyre structure over the North Pacific, the residual trend shows a double-gyre pattern over the Northeast Pacific region, which resembles the NPGO-winds in this region (compare Figs. 2e, f with 6a, b).

An ocean general circulation model (OGCM) experiment with surface wind and heat flux forcing (freshwater flux being fixed to climatology) reasonably reproduced the observed sea level variability over the Northeast Pacific region for the 1950–2004 period, and salinity variability in this experiment results from advection rather than from forcing by freshwater fluxes (Di Lorenzo et al. 2008). Independently, Bromirski et al. (2011) also examined the effects of surface wind and heat flux using OGCM experiments, showing that local wind stress curl dominates heat flux in causing decadal sea level variability along the US west coast. Thompson et al. (2014) suggested the dominance of remote equatorial wind stress, rather than the local wind stress curl, in driving decadal sea level variability along the US west coast during recent decades. These results suggest that the decadal sea level patterns associated with the PDO and NPGO, which are dominated by the upper-ocean thermosteric sea level (e.g., Lombard et al. 2005), result primarily from changes in wind-driven ocean circulation, with surface heat and freshwater fluxes playing a minor role in the North Pacific Ocean. Indeed, the dominance of wind-driven ocean circulation in causing regional distributions of decadal sea level variability has been demonstrated, and the thermosteric and halosteric sea level components often have compensating effects due to heat and salt redistribution by advection [see reviews by Stammer et al. (2013) and Kopp et al. (2015)].

Diabatic fluxes, however, are shown to have significant contributions to the 1993–2004 trends of decadal sea level—particularly thermosteric sea level—in the western tropical Pacific Ocean (Fukumori and Wang 2013). Forcing by surface buoyancy flux is also shown to be non-negligible in causing interannual sea level variability in the tropical south Pacific and subtropical North Pacific, and the effect can be non-local due to advection and Rossby wave propagation (Piecuch and Ponte 2012; Forget and Ponte 2015). These results suggest that heat and freshwater fluxes associated with the PDO and NPGO may also induce significant decadal sea level variability in some regions. This aspect requires further study.

Note that even though the importance of winds has been emphasized, and PDO- and NPGO-related surface wind patterns have been extracted through regression analyses (Figs. 2, 3, 6), model experiments that use the extracted winds as surface forcing to quantify the roles played by the PDO and NPGO have not yet been performed. Moreover, the regression method may not cleanly isolate the wind signals associated with the climate modes (Palanisamy et al. 2015), and reanalysis winds that were used in the regression analyses have significant uncertainties (e.g., Wittenberg 2004), with apparent discrepancies or even opposite signs in their multidecadal trends since the 1960s (Nidheesh et al. 2013). Yet, reanalysis winds have been widely applied to investigate decadal climate variability due to their longer data records than satellite winds, and decadal trends of sea level over tropical oceans are very sensitive to the wind trends (McGregor et al. 2012). These issues will be further discussed in Sect. 6.

## The Indian Ocean

### Changes in Walker and Hadley Circulations and Related Sea Level Trend Patterns

**Fig. 7:**
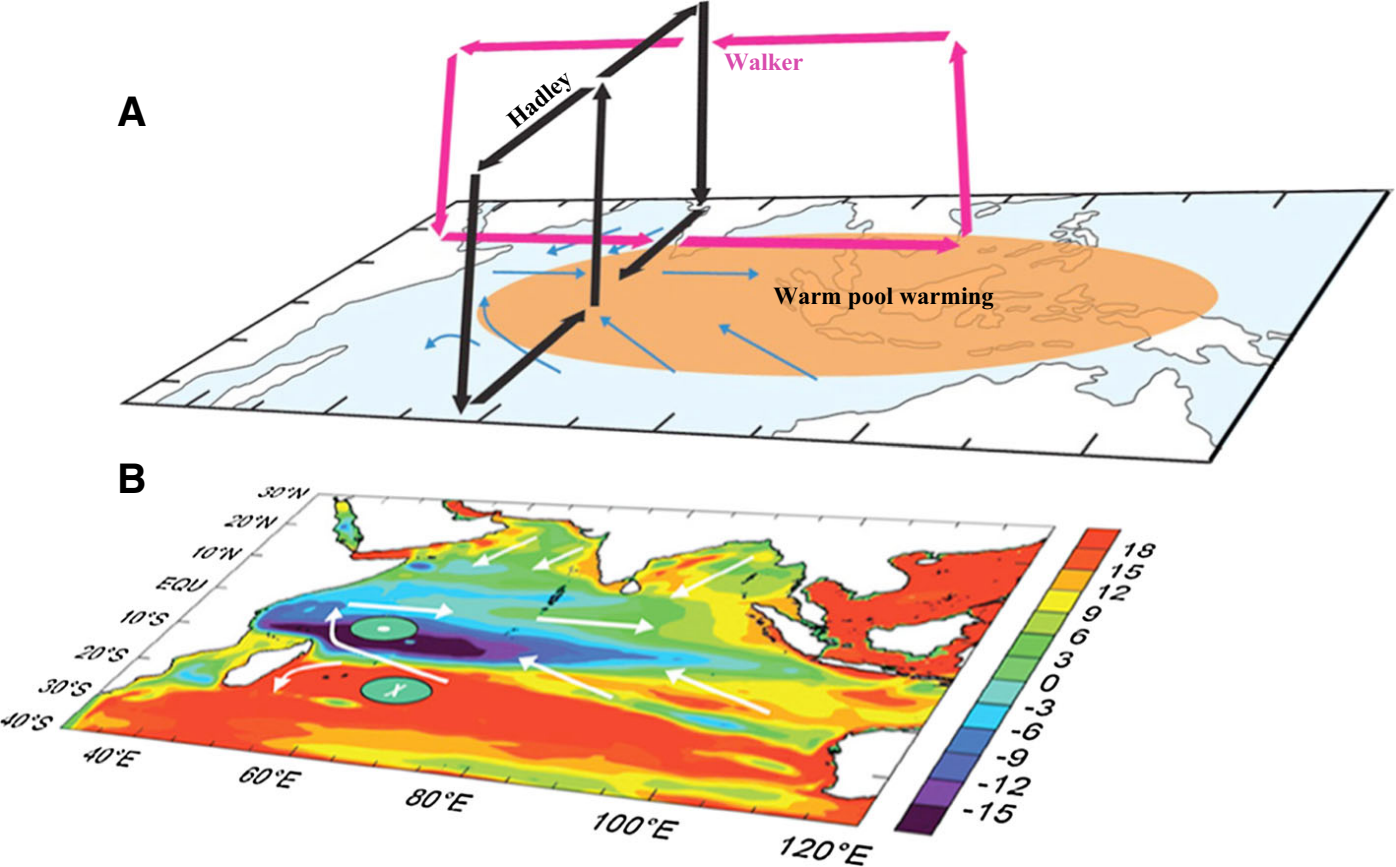
A schematic diagram showing the mechanisms for the Indo-Pacific warm pool warming to cause the Indian Ocean (IO) sea level change. Warming enhances the IO regional Hadley and Walker cells (**a**); the two enhanced cells combine to form a specific pattern of surface wind change (surface *arrows* in **a** and **b**) together with the Ekman pumping velocity (positive—*circle* with *dot*; negative—*circle* with *x*), which drive the distinct sea level pattern (*color* contours in **b**). From Han et al. (2010)

The Indian Ocean sea level trends since the 1960s (from 1961 to 2001) exhibit a basin-wide pattern, with sea level falling in the southwest tropical basin and rising elsewhere (Fig. 7b) (e.g., Han et al. 2010; Timmermann et al. 2010; Dunne et al. 2012; NRC 2012).

Experiments using an OGCM and an atmospheric GCM (AGCM) show that surface winds associated with enhanced Indian Ocean Walker and Hadley circulations are the major force for this basin-wide pattern (Fig. 7) (Han et al. 2010). Variation of the Indonesian Throughflow may have a significant contribution to the thermocline cooling and thus sea level fall in the southwest tropical Indian Ocean (Schwarzkopf and Böning 2011), as shown by OGCM experiments forced with different reanalysis winds from that of [Han et al. (2010); see Han et al. (2014b) for a review]. The “basin-wide patterns” of surface warming and thermocline cooling, however, are still mainly caused by Indian Ocean winds in the Schwarzkopf and Böning (2011) study (see their Fig. 2).

While the multidecadal trends of surface winds associated with the changing Walker and Hadley circulations are shown to be partly forced by the Indian Ocean warming (Han et al. 2010), which is attributed primarily to anthropogenic forcing since the 1950s (Du and Xie 2008; Dong et al. 2014), they are suggested to have a large contribution from natural climate variability (Timmermann et al. 2010). What natural climate modes account for the changes of Indian Ocean Walker and Hadley circulations, and what role does the changing Indian monsoon play? These important science issues remain to be explored.

### Climate Modes, Sea Level Patterns and Processes

Overlying the multidecadal trend, satellite observations show large decadal variations of the basin-scale sea level patterns, with reversing trends from 1993 to 2000 and 2000 to 2006 (Lee and McPhaden 2008). Over the North Indian Ocean (north of 5°S), sea level experienced basin-wide falls from 1993 to 2003 but sharp rises from 2004 to 2013 (Srinivasu et al. 2016, Climate Dynamics, revised; Thompson et al. 2016). However, similar reversals did not occur over the south Indian Ocean (south of 5°S), the Pacific and the Atlantic Oceans. Both observational analyses and OGCM experiments suggest that winds over the Indian Ocean are the primary forcing for the basin-wide decadal sea level patterns (e.g., Trenary and Han 2013; Nidheesh et al. 2013; Zhuang et al. 2013), with the Indonesian Throughflow having a significant contribution primarily in the eastern basin (e.g., Trenary and Han 2013). A similar conclusion also holds for intraseasonal-to-interannual sea level variability over the south Indian Ocean (Trenary and Han 2012). The observed North Indian Ocean basin-wide decadal reversal of sea level results from the combined effect of changing surface turbulent heat flux and the cross-equatorial heat transport, with both being associated with decadal changes of surface winds (Srinivasu et al. 2016, Climate Dynamics, revised; Thompson et al. 2016). Thermosteric sea level is the primary contributor for the spatial patterns of decadal sea level variability (e.g., Fukumori and Wang 2013; Nidheesh et al. 2013), with halosteric sea level having apparent contributions in some regions (Shankar and Shetye 1999; Nidheesh et al. 2013) particularly in the southeast tropical Indian Ocean and near the west Australian coast in the past decade (Llovel and Lee 2015).

#### Effects of Climate Modes and Processes

**Fig. 8:**
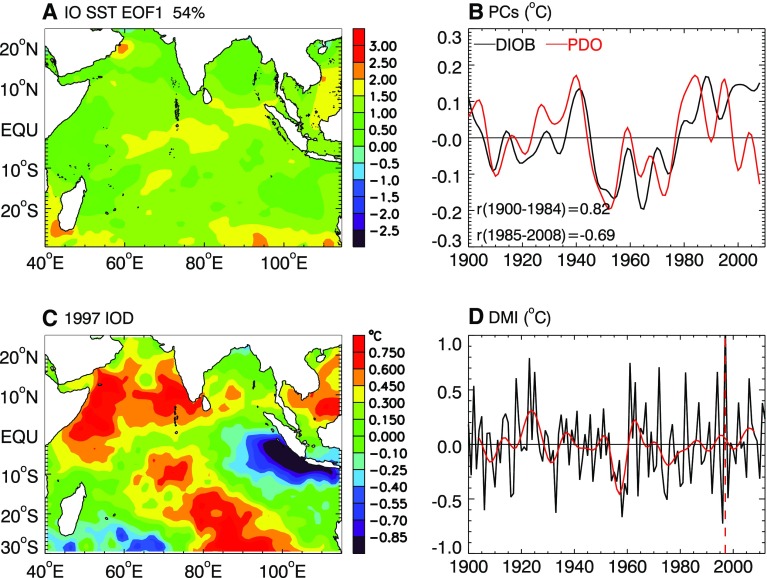
**a** The leading EOF of SST anomaly (SSTa) for the Indian Ocean, based on 8-year low-pass-filtered monthly HadISST from 1900 to 2008, which explains 54 % variance and is referred to as the Decadal Indian Ocean Basin Mode (DIOB), **b** The PC1 of 8-year low-passed SSTa (*black*; DIOB index) and North Pacific SST PC1 (*red*; PDO index), **c** September, October and November mean SSTa for the 1997 IOD event, based on the detrended and demeaned SST from 1900 to 2012, **d** Dipole Mode Index (DMI; *black*) for each year, which is defined as the September, October and November mean SSTa difference between the western pole (50°E–70°E, 10°S–10°N) and eastern pole (90°E–110°E, 10°S–0°N); the *red curve* is the 8-year low-passed DMI, which shows decadal variability. *Dashed vertical line* marks the 1997 IOD

To what extent are the observed patterns of decadal sea level induced by climate modes? Over the tropical Indian Ocean, two climate modes have been identified in SST anomaly (SSTa): the Indian Ocean basin mode and Indian Ocean Dipole (IOD). The decadal SSTa is dominated by the decadal Indian Ocean basin mode (DIOB), defined as the leading EOF of decadal SSTa with a basin-wide warming/cooling pattern and explaining 54 % of the variance (Fig. 8a) (Han et al. 2014b). The DIOB index, defined as PC1 of SSTa, is positively correlated with the PDO before 1985 (*r* = 0.82; Fig. 8b), similar to the ENSO influence on Indian Ocean interannual SSTa (Klein et al. 1999; Xie et al. 2009). The correlation, however, reverses to become negative after 1985 (*r* = −0.69; Fig. 8b) (Han et al. 2014a). Causes for this change in character remain unclear, but the negative correlation indicates that Indian Ocean internal processes—likely related to monsoon variability—may be important for generating the DIOB. The IOD is an interannual coupled ocean–atmosphere climate mode (e.g., Saji et al. 1999; Webster et al. 1999), and its positive phase is associated with cold SSTa in the eastern tropical Indian Ocean and warm SSTa in the western tropical basin (Fig. 8c), reaching peak amplitudes during September–November. Its temporal variability is measured by the Dipole Mode Index (DMI; Fig. 8d), which exhibits large interannual variability with decadal modulation (black and red curves of Fig. 8d) (see also Ashok et al. 2004; Song et al. 2007; Tozuka et al. 2007 for IOD decadal variability).

Li and Han (2015) performed a suite of experiments using an eddy-permitting (0.25° × 0.25°) OGCM over the Indian Ocean basin for the 1948–2012 period, to assess the roles played by surface wind stress associated with climate modes, stochastic wind stress forcing and surface heat and freshwater fluxes. To extract the wind stress signals associated with the DIOB and IOD, Li and Han (2015) first performed multiple linear regression onto the observed Multivariate ENSO Index (MEI) and DMI, using National Center for Environmental Prediction (NCEP) reanalysis winds and SST data from HadISST (Hadley Centre Sea Ice and Sea Surface Temperature data set). The wind stress and SST anomalies regressed onto MEI account for part of the DIOB because it is highly correlated with ENSO (or PDO) before 1985. Then, they employed a singular value decomposition (SVD) technique to obtain the covariance of the residual wind stress and SST with ENSO and IOD signals removed. The leading 7 SVD modes were obtained, which include the part of the DIOB that is independent of PDO. The sum of wind stress anomalies related to ENSO, IOD and the SVD modes measure the total effects of Indian Ocean climate modes. Experiment results of Li and Han (2015) show that the basin-wide decadal sea level patterns over the tropical Indian Ocean (north of 20°S) are forced mainly by wind stress associated with climate modes, with the maximum amplitude occurring in the southwest tropical Indian ocean thermocline ridge region (their Figs. 4, 6). Surface heat flux has a significant contribution in the subtropical basin between 20°S and 28°S, consistent with Fukumori and Wang (2013) for the effect of diabatic fluxes on 1993–2004 sea level trend in this region. Stochastic wind stress forcing has a large contribution in the southwest Indian Ocean (south of 30°S).

#### DIOB-Related Sea Level Patterns

Even though Li and Han (2015) demonstrated the dominant influence of climate modes on sea level in the tropical Indian Ocean, basin-wide sea level patterns associated with the DIOB alone, including both PDO influence and internal coupled processes related to monsoon variability, have not yet been assessed. Intuitively, the basin-wide warming and cooling associated with the DIOB will produce basin-wide sea level rise or fall due to thermal expansion. Changes in ocean circulation associated with the DIOB, however, remain unknown. Given that DIOB is significantly correlated with PDO before 1985, Indian Ocean sea level patterns associated with the PDO based on reconstructed sea level for 1950–2010 (Fig. 1a) and Simple Ocean Data Assimilation (SODA) product for 1958–2007 (second row in left column of Fig. 3) reflect a portion of sea level patterns associated with DIOB. The patterns from the two datasets over the Indian Ocean, however, show significant differences, indicating uncertainties involved with different datasets.

#### IOD-Related Sea Level Patterns

**Fig. 9:**
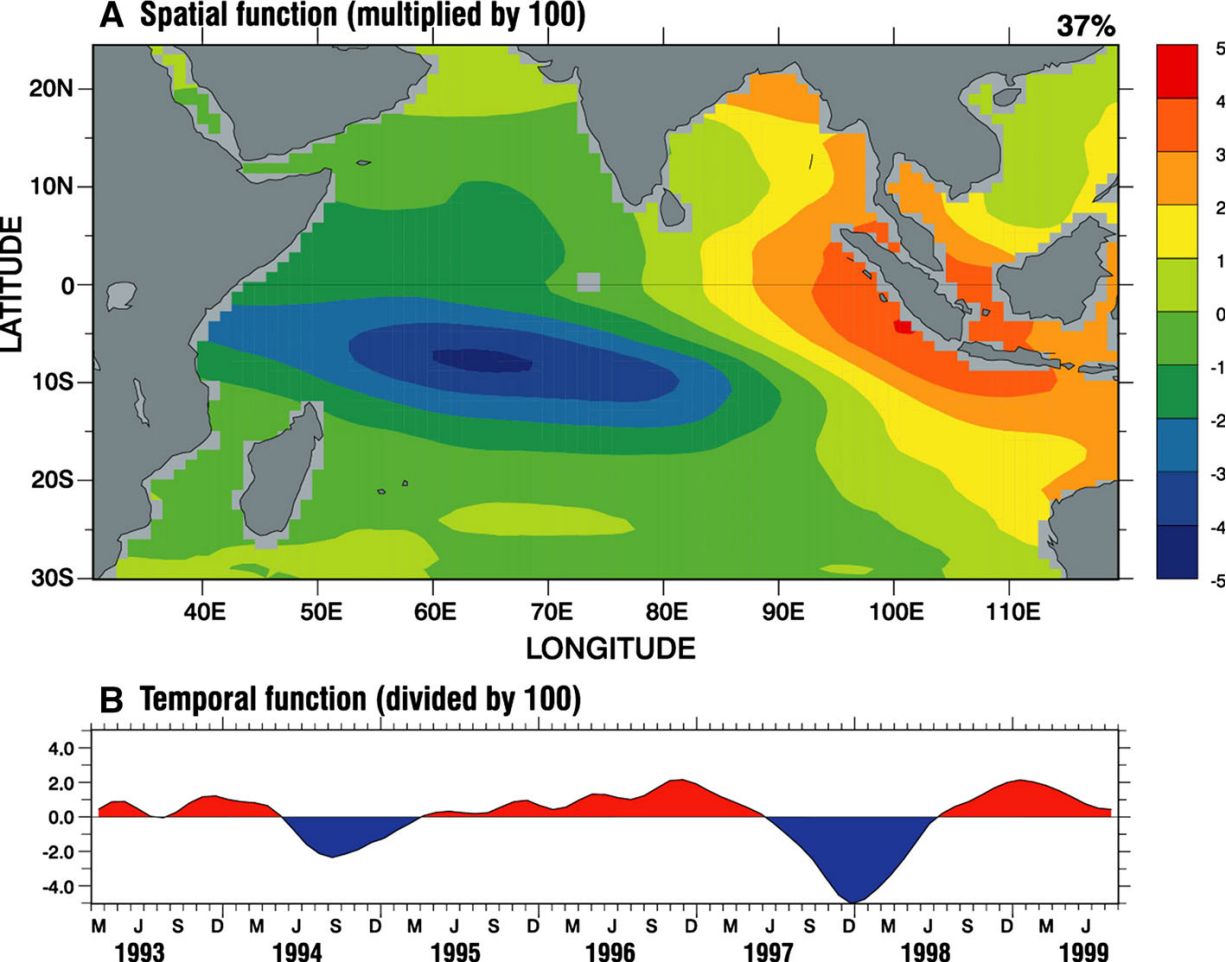
The leading Complex EOF of SSH (**a**) and PC1 (**b**), based on Topex/Poseidon SSH anomaly from 1993 to 1999 (downloaded from: http://www.jamstec.go.jp/frcgc/research/d1/iod/fig_1.jpg)

The spatial patterns of sea level associated with a negative IOD show SLR in the eastern basin, with the maximum amplitude being located at the equatorial basin and extending northward into the Bay of Bengal and southward to the west coast of Australia, which accompanies sea level fall in the western tropical basin with the maximum amplitude occurring in the Seychelles-Chagos thermocline ridge region (Figure 2 of Han and Webster 2002). These spatial patterns resemble that of SSHa EOF1 (Fig. 9a) (Rao et al. 2002), which represents the SSHa patterns associated with the IOD because its PC1 (Fig. 9b) is highly correlated with the DMI (Rao et al. 2002). The EOF1 patterns shown in Fig. 9 are similar to the regression patterns with DMI (fifth row of Fig. 3). The large SSHa amplitudes in the eastern basin and in the thermocline ridge region, together with the minimum amplitudes in the central equatorial basin and eastern Arabian Sea, agree with the patterns of interannual SSHa variance shown by Shankar et al. (2010). These results suggest that, under periodical wind forcing with periods longer than 17 months, sea level variability over the tropical Indian Ocean (north of 10°S) is in quasi-steady balance with wind variability, and the sea level maxima (minima) result from the constructive (destructive) interference between directly forced response and Rossby waves reflected from the eastern boundary (Shankar et al. 2010).

Frankcombe et al. (2015) showed significant decadal variations in the relationship between IOD and sea level patterns by analyzing OGCM results and SODA data: the regression coefficients are enhanced over the Indian Ocean since the 1990s compared to the preceding decades (their Figs. 6, 7). By analyzing historical SST and reanalysis data, it has been shown that IOD events exhibit an increasing trend in frequency and strength during the twentieth century (Abram et al. 2008), and positive IOD events prevail after 1950, which corresponds to an upward trend of the IOD index (e.g., Kripalani and Kumar 2004; Ihara et al. 2008; Yuan et al. 2008; Cai et al. 2009; Fig. 8d). This upward trend, however, appears in some SST datasets but not in others, highlighting the uncertainties in these trends (Cai et al. 2013). With frequently occurring intensified positive IOD events, we expect enhanced and more frequent sea level rise in the thermocline ridge region and sea level fall in the eastern basin during boreal fall (Fig. 9), which may counteract the multidecadal sea level trend patterns (Fig. 7) in many regions.

#### Subtropical Dipole and Sea Level Patterns

In the south Indian Ocean, a subtropical dipole has also been identified in interannual SSTa with peak development in austral summer (Behera and Yamagata 2001; Suzuki et al. 2004). A positive phase is characterized by warm SSTa in the southwestern Indian Ocean south of Madagascar and cold SSTa in the eastern Indian Ocean off Australia. It also undergoes decadal variations, showing weakened amplitudes after 1979–1980 (Yan et al. 2013). Compared to other climate modes, few studies have investigated the influence of the subtropical dipole on sea level. Thompson et al. (2016) showed that periods of enhanced cross-equatorial overturning circulation in the Indian Ocean tend to occur during the positive subtropical dipole phase when the Mascarene High sea level pressure is strengthened and shifted to the north. The shift of this prominent atmospheric circulation alters wind stress curl, which drives changes in the overturning and affects heat content and sea level north of the equator.

## The Atlantic

### Sea level Variability, Forcing and Relation to Changes of Atlantic Meridional Overturning Circulation (AMOC) and the Gulf Stream

Tide gauge and satellite observations show significant interannual-to-decadal sea level variability over the Atlantic during the past several decades and century (e.g., Figure 1 of Kenigson and Han 2014). Observational analyses combined with model experiments have been carried out to understand their causes. In the western North Atlantic and along the US east coast, existing studies suggest the dominance of wind stress curl over the basin interior in driving westward-propagating Rossby waves, affecting interannual and decadal (periods >3 years) sea level variability from 18°N to 38°N (e.g., Sturges and Hong 1995; Hong et al. 2000; Thompson and Mitchum 2014). The regional along-shelf wind stress is shown to be important for interannual sea level variability from Nova Scotia to North Carolina (e.g., Andres et al. 2013; Woodworth et al. 2014). Near the eastern boundary of the North Atlantic, coherent decadal sea level variability has been observed during the past century (e.g., Kolker and Hameed 2007; Miller and Douglas 2007; Woodworth et al. 2010; Sturges and Douglas 2011; Calafat et al. 2012), and it has been attributed to forcing by local longshore winds and coastal wave propagation (e.g., Sturges and Douglas 2011; Calafat et al. 2012), with mass redistribution having a small contribution (Calafat et al. 2012). This result is in contrast to Woodworth et al. (2010), who suggested the importance of mass redistribution in affecting east Atlantic coastal sea level.

**Fig. 10:**
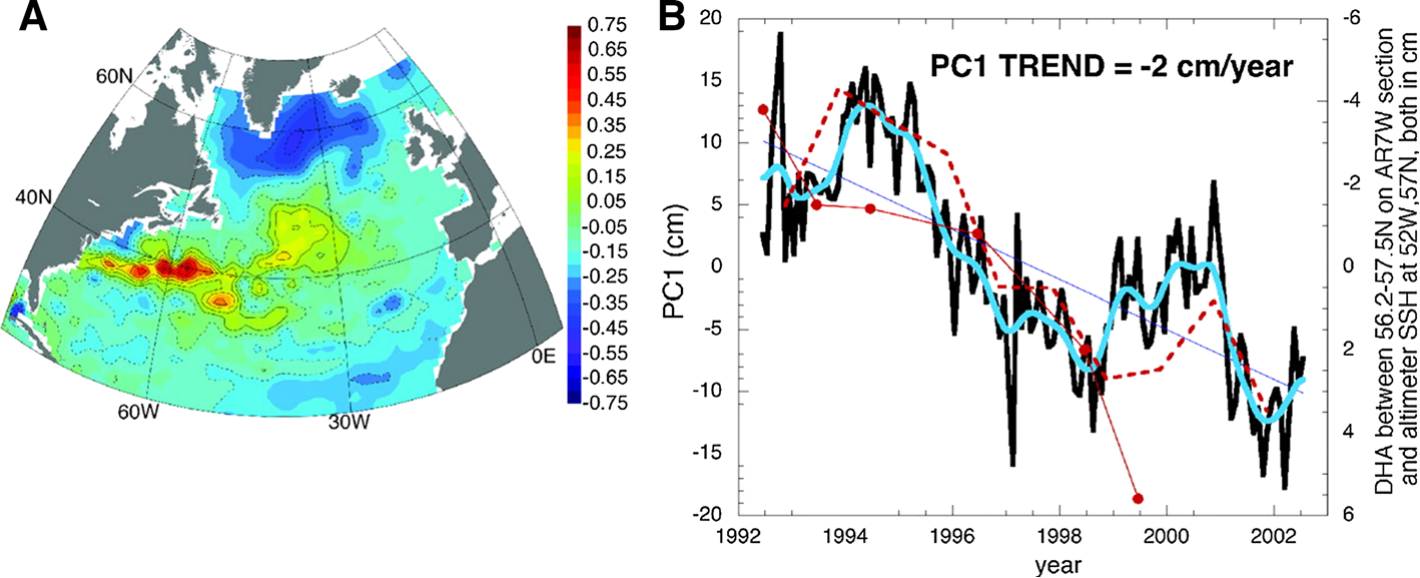
(*Left*) EOF1 of altimeter SSH and (*right*) its PC1 (*black curve*). The *blue curve* is the binomially smoothed PC1. The dynamic height anomaly (DHA; in *solid red*; *dots* denote data points of the time series) computed in the central Labrador Sea (average from 56.2° to 57.5°N along the WOCE AR7/W section across the Labrador Sea from Newfoundland to Greenland) is shown in the *right panel* with its axis on *right*. The altimeter SSHa at 52°W, 57°N (12-month May-to-April average) is shown in *dashed red curve*. Adapted from Hakkinen and Rhines (2004)

In the North Atlantic basin interior, OGCM experiments spanning 1951–2000 demonstrate that decadal variability in the leading EOF modes of SSHa and gyre circulation originate from the basin-scale thermal forcing, rather than from wind stress driving, and that low-frequency variations of SSH along the Gulf Stream reflect predominantly the Atlantic Meridional Overturning Circulation (AMOC) changes (Hakkinen 2001). The importance of buoyancy and mass forcing in affecting interannual variability of sea level through advection and Rossby wave propagation in the tropical South Atlantic and subtropical to subpolar North Atlantic has also been suggested (Piecuch and Ponte 2013; Forget and Ponte 2015). The leading EOF mode of satellite-observed or model-simulated SSHa exhibits a dipole pattern (Fig. 10): with low SSHa occurring in the Subpolar Gyre region (extending southwestward to the US northeast coast) being accompanied by high SSHa in the Subtropical Gyre region. Its PC1 shows large-amplitude decadal variability (Figs. 2, 3, 4 of Hakkinen 2001). This SSH dipole resembles the distinctive fingerprint of AMOC variability (Figure 1 of Zhang 2008), except that the extension to the US Northeast coast from the Subpolar Gyre disappears in the AMOC fingerprint. This difference may indicate the importance of local winds in driving coastal sea level particularly north of Cape Hatteras, as suggested by recent studies (Andres et al. 2013; Woodworth et al. 2014).

Recently, accelerated SLR along the US northeast coast, particularly in the “hotspot” from Cape Hatteras to Cape Cod since the 1950s, has been detected using tide gauge observations (Sallenger et al. 2012). The weakened transport of the Gulf Stream, the upper branch of the AMOC, and the northward shift of the Gulf Stream during recent decades (Ezer 2013; Ezer et al. 2013; Yin and Goddard 2013) have been suggested to be the cause. The Gulf Stream sustains a sharp sea level gradient associated with geostrophic balance, suppressing sea level along the Atlantic coast by 1–1.5 m relative to the open ocean east of the Stream. A weakened Gulf Stream, therefore, weakens offshore sea level gradients and causes SLR along the US east coast. Furthermore, a weakened AMOC (e.g., due to surface warming and Greenland Ice Sheet melting) can induce rapid SLR along the US northeast coast and in the Subpolar Gyre region, based on OGCM and climate model simulations (e.g., Bingham and Hughes 2009; Yin et al. 2009; Hu et al. 2009). Andres et al. (2013), however, argued that the changes observed along the coast and over the shelf appear to influence the Gulf Stream path downstream of Cape Hatteras, rather than the changes in Gulf Stream transport affecting coastal sea level. Woodworth et al. (2014) analyzed the results from data-assimilative OGCM experiments (also see Williams et al. 2014) with and without wind stress forcing for the 1950–2009 period and suggested that although a relationship between US northeast coast sea level change and the AMOC variation can be identified (an increase of ~1.5 cm in sea level for a decrease of 1 Sv in MOC transport), it is the surface wind stress particularly the regional wind over the shelf that dominates the sea level variability along the US northeast coast.

### NAO-Related Sea Level Patterns

Are the interannual and decadal sea level variations over the Atlantic Ocean associated with the known climate modes? Indeed, many studies have linked regional or basin-scale sea level variability, surface wind and heat flux to the North Atlantic Oscillation (NAO). These studies use EOF analysis by comparing the PCs with the NAO index or calculate correlation coefficients between the NAO index and regional sea level variability. The NAO is a major atmospheric circulation anomaly pattern corresponding to fluctuations in Icelandic low sea level pressure (SLP) in the north and Azores high SLP to the south (e.g., Barnston and Livezey 1987; Hurrell 1995 and references therein). The winter (December–March) station-based NAO index is measured by the difference of normalized SLP between Azores high and Icelandic low (for NAO index and spatial patterns, see https://climatedataguide.ucar.edu/climate-data/hurrell-north-atlantic-oscillation-nao-index-station-based). A positive NAO phase corresponds to a positive SLP anomaly in Azores high and a negative SLP anomaly in Icelandic low. Note that variations in both buoyancy flux and surface wind stress, which in part are associated with the NAO, can induce changes in AMOC (e.g., Danabasoglu 2008; Schloesser et al. 2012, 2014; Yeager and Danabasoglu 2014) and thus affect SLR in the Subpolar Gyre, Subtropical Gyre, and likely along the US northeast coast.

A distinct 12–14-year spectral peak appears in all tide gauge stations along the US east coast from Charleston to Eastport with record lengths at least from 1930 to 2012 (Figure 1 of Kenigson and Han 2014). Hakkinen (2000) suggested that the 12–14-year cycles of US east coast sea level and basin-scale SST result mainly from surface heat flux forcing, whose leading EOF is associated with the NAO; but this does not exclude forcing of freshwater flux from enhancing or weakening the cycle. The author argued that the 12–14-year cycle is potentially a coupled ocean–atmosphere mode: Starting from the positive NAO phase, positive SST and oceanic heat content anomalies in the subtropics are advected to the Subpolar Gyre, where they are amplified by local heat flux, a positive feedback between the atmosphere and ocean. Meanwhile, this warm advection causes a negative feedback of the AMOC on itself, which is amplified by the positive feedback between the atmosphere and ocean in the Subpolar Gyre. As a result, the AMOC slows down and the opposite cycle starts (Hakkinen 2000). Andres et al. (2013) argued that the NAO has a strong influence on interannual variability of sea level along the US northeast coast from 1987 to 2012 but not during 1970–1987 (their Fig. 3a). Ezer (2013) and Ezer et al. (2013) suggested that the weakening Gulf Stream and AMOC, which affect the hotspot SLR acceleration during recent decades, are linked to the Atlantic Multidecadal Oscillation (AMO) but are also affected by the SLP and winds associated with the NAO. The effects of NAO-related wind stress curl and surface heat flux in the basin interior on interannual and decadal variability of the Gulf Stream have also been suggested by other studies (e.g., Curry and McCartney 2001; Di Nezio et al. 2009; Chaudhuri et al. 2011). This effect is shown to be important from 1986 to 1998 compared to the periods before and after (Meinen et al. 2010). Indeed, the 2009–2010 extreme SLR along the US northeast coast has been attributed to a strong negative NAO superimposed on a 30 % AMOC reduction (Goddard et al. 2015). Note that the inverted barometer (IB) effect accounts for approximately 50 % of the extreme SLR event (Piecuch and Ponte 2015).

**Fig. 11:**
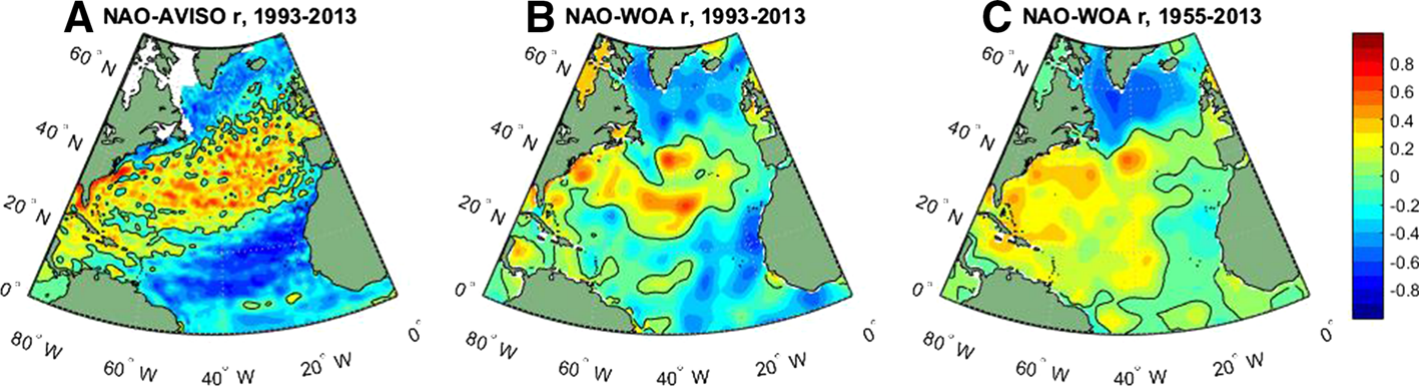
**a** Correlation map between winter (DJFM mean) NAO index and annual mean AVISO satellite SSHa over the North Atlantic Ocean for the 1993–2013 period; zero correlation values are shown by the *black line contours*, **b** Same as **a** but for NAO and WOA13 upper 700 m thermosteric sea level (Levitus et al. 2012) from 1993 to 2013; **c** Same as **b** but for the 1955–2013 period. The global mean SLR is removed from each panel before the correlation calculation (The NAO index is downloaded from: www.climatedataguide.ucar.edu/climate-data/hurrell-north-atlantic-oscillation-nao-index-station-based)

Hakkinen and Rhines (2004) observed a downward trend of the Subpolar Gyre during the 1990s, which corresponds to SLR in the Subpolar Gyre and along the US northeast coast and sea level fall in the Gulf Stream and Subtropical Gyre (Fig. 10). They suggested that buoyancy forcing associated with the NAO is the major cause. The SSH patterns of Fig. 10 resemble the correlation map between the NAO index and satellite-observed SSHa from 1993 to 2013 (Fig. 11a) and the EOF1 patterns based on the observed upper 500 m thermosteric sea level from 1950 to 1998 (Lombard et al. 2005). The PC1 is significantly correlated with the NAO index with a correlation coefficient of 0.55, and the two agree well particularly on decadal timescales (Lombard et al. 2005). The EOF1 patterns of Lombard et al. (2005) are similar to the correlation map between the NAO index and the WOA13 upper 700 m thermosteric sea level from 1955 to 2013 (Fig. 11c), and to the spatial structure of upper 500 m temperature anomalies during a period of rapid change in the sea level circulation index of McCarthy et al. (2015). Note that there is a major difference between Fig. 11a, b: the correlation coefficients along the US northeast coast have opposite signs. This difference likely results from the lack of in situ observations near the coasts and the coarser 1° × 1° resolution of thermosteric sea level data compared to the 0.25° × 0.25° resolution AVISO data, which can better resolve sea level variability near the coast (compare Fig. 11a, b). Lozier et al. (2010) showed that changes in surface winds associated with the upward trend of NAO from 1950 to 2000 (Hurrell 1995) enhanced the mean AMOC strength over the Subpolar Gyre latitudinal band but weakened the AMOC in the Subtropical Gyre latitudinal band, suggesting that the AMOC changes can be gyre specific instead of a single, basin-scale overturning cell.

Over the Northeast Atlantic and west coast of Europe, decadal sea level variability is also significantly correlated with the NAO. Variations of SLP via IB effect and large-scale winds associated with the NAO are probably the cause, with wind stress playing a more important role (Miller and Douglas 2007; Calafat et al. 2012). Kolker and Hameed (2007) showed that changes in the center of action of the Icelandic low and Azores High play a crucial role in affecting decadal sea level variability and multidecadal trend along both the west and east coasts of the North Atlantic Ocean. The shift in the NAO center of action can affect surface winds, SLP and SST, thereby influencing sea level via a suite of coastal oceanographic processes. This result is important, indicating that the effects of NAO can be reflected in both the NAO index—which measures its strength variations—and in the location shift of its center of action. The relationship between sea level in the eastern North Atlantic and the NAO has also been demonstrated by earlier studies (e.g., Wakelin et al. 2003; Woolf et al. 2003; Yan et al. 2004; Tsimplis et al. 2006; Tsimplis and Shaw 2008). They suggested that the relationship is spatially and temporally variable, with stronger NAO influence during the second half of the twentieth century than the first half.

### Atlantic Multidecadal Oscillation (AMO)-Related Sea Level Patterns

The AMO is defined as a mode of natural variability with coherent warming or cooling in the North Atlantic Ocean and an estimated period of 60–80 years (Schlesinger and Ramankutty, 1994; Delworth and Mann 2000; Kerr 2000; Enfield et al. 2001). The AMO index is based upon the detrended average SST anomalies in the North Atlantic, typically over 0–80°N (Trenberth and Shea 2006). Since 1940, the AMO index shows a negative transition (cooling trend) from the 1940s to 1970s and positive transition (warming trend) afterward, forming a ~60-year cycle with negative phase from the 1960s to 1990s. Surface warming over the North Atlantic during recent decades has been suggested to reflect partly anthropogenic forcing and partly positive AMO transition (Ting et al. 2009). By contrast, several recent studies suggest that the ~60-year oscillation in the AMO index in the past few decades can be externally forced by aerosols (natural and anthropogenic), volcanic eruptions, and the solar cycle (e.g., Booth et al. 2012; Knudsen et al. 2014). However, Zhang et al. (2013) argued that external forcing might not be the dominant causative factor. Mann et al. (2014) suggested that the “detrending method” failed to isolate the true internal variability, and the true AMO signal is likely to have been in a cooling phase during recent decades. Due to this controversy, in our discussion below we refer to the AMO index of Trenberth and Shea (2006) as “the ~60-year cycle of SST index.”

Tide gauge observations detected robust accelerations of SLR along the highly populated US northeast coast since 1950 and especially since 1970 (Sallenger et al. 2012; Boon 2012; Ezer and Corlett 2012). Existing studies suggest that the ~60-year cycle in sea level, which is present in most tide gauge stations along the US northeast coast (Kenigson and Han 2014) with rapid SLR since 1970 coinciding with the positive transition of the ~60-year cycle of SST index, has a significant contribution to the observed SLR acceleration (Kopp 2013; Ezer 2013; Scafetta 2014; Kenigson and Han 2014). Using both Empirical Mode Decomposition (EMD) and ensemble EMD methods, Kenigson and Han (2014) constructed synthetic tide gauge data by extracting the leading oscillations at interannual-to-multidecadal timescales from tide gauge data, and extended the data back to 1813 by superimposing the oscillations on prescribed trends with known acceleration rates. Experiments with and without the ~60-year cycle demonstrated that the ~60-year cycle has indeed contributed a significant portion to the hotspot acceleration since 1970, and a record length of approximately twice the ~60-year period (see also Scafetta 2014) is required in order to adequately detect the long-term, nonlinear acceleration rate with errors <25 %.

By examining long tide gauge records during the twentieth century, Chambers et al. (2012) showed that a ~60-year cycle appears in the majority of tide gauge data over the Atlantic, Indian and Pacific Oceans. They suggested that there is a possibility that the ~60-year oscillation is present in global mean sea level, even though the tide gauge data are still too limited in time and space to make a definitive conclusion. In fact, Jevrejeva et al. (2008) found a ~60-year cycle in the global mean sea level of their reconstructed data since 1700 and speculated that it might be associated with the AMO.

What are the spatial patterns of decadal sea level variability induced by the natural internal AMO mode of the climate system? This issue has not yet been addressed. Note that the AMO can affect the North Atlantic SLR in two ways: The basin-wide warming during a positive AMO phase can increase sea level by thermal expansion, and the enhanced AMOC associated with a positive AMO (see review by Liu 2012 for MOC mechanisms and references therein) tends to weaken the US northeast coast and Subpolar Gyre SLR (e.g., Hu et al. 2009; Yin et al. 2009). The two effects combine to yield the spatial patterns of sea level change associated with the AMO. Kopp (2013) argued that the combination of the two effects produces a positive correlation between hotspot interdecadal SLR and AMO index. As discussed above, however, the oscillation of SST index in the past few decades may be externally forced and thus its positive correlation with the hotspot sea level may not reflect the true internal AMO mode.

**Fig. 12:**
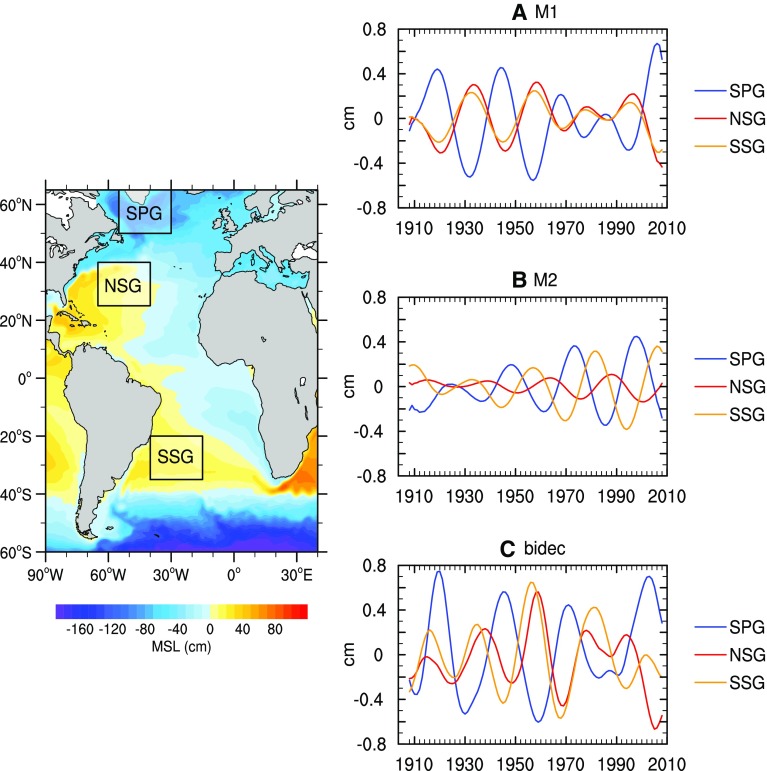
Three space-averaged SSHa indices for the **a** EOF1 (M1), **b** EOF2 (M2) and **c** the bidecadal band, computed for each of the three boxes (SPG, NSG and SSG) shown on the map. SPG stands for Subpolar Gyre, NSG for North Subtropical Gyre, and SSG for South Subtropical Gyre. *Color contours* in the *map* show the 1908–2008 mean sea level from SODA data. The *map* is only used to illustrate the locations of the *boxes*. From Vianna and Menezes (2013)

Several studies have shown prominent bidecadal (20–30-year) variability in SST, SLP and sea level over both the North and South Atlantic (e.g., Venegas et al. 1998; Danabasoglu 2008; Frankcombe and Dijkstra 2009; Chylek et al. 2011; Vianna and Menezes 2011, 2013). While most observational and modeling studies suggest that the bidecadal oscillations can be largely explained by internal ocean dynamics related to the AMOC and AMO (Huck and Vallis 2001; Von der Heydt and Dijkstra 2007; Danabasoglu 2008; Frankcombe and Dijkstra 2009; Frankcombe et al. 2010), others argue that they involve westward-propagating baroclinic Rossby waves of large-scale temperature and sea level anomalies (e.g., Sevellec and Fedorov 2013; Vianna and Menezes 2013). Sevellec and Fedorov (2013) demonstrated that the zonal structures of temperature anomalies alternate between a dipole (corresponding to AMOC variability) and one sign pattern (no AMOC variability). Consistent with this result, Vianna and Menezes (2013) showed that the leading CEOF mode of the bidecadal sea level signals is associated with the AMOC variability, whereas the second CEOF mode has distinguishable westward-propagating thermal Rossby waves and is not apparently related to AMOC change. The leading CEOF mode is characterized by in phase North and South Subtropical Gyres with an opposite sign in the tropical and subpolar regions (Fig. 12a), and it dominates sea level variability from 1915 to 1965 (Fig. 12). For the CEOF2 mode, the North and South Subtropical Gyres are not in phase (Fig. 12b) and it dominates the bidecadal sea level after 1970 (compare Fig. 12a–c) (also see Figure 1 of Vianna and Menezes 2013). These results indicate that patterns of sea level can vary on multidecadal timescales, corresponding to the time-varying effects of different oceanic processes (e.g., AMOC vs. Rossby waves).

## The Arctic and Southern Oceans

### The Arctic Ocean

The Arctic Oscillation (AO) is a large-scale pattern of climate mode characterized by SLP over the polar region varying in opposition to that over middle latitudes (about 45°N) on time scales ranging from weeks to decades (e.g., Thompson and Wallace 1998). The AO is also referred to as “Northern Annular Mode,” and the NAO is its North Atlantic expression (http://nsidc.org/arcticseaicenews/tag/arctic-oscillation/). The AO index is constructed by projecting the daily 1000 mb height anomalies poleward of 20°N onto the loading pattern of the AO, which is the leading EOF of monthly mean 1000 mb height anomaly (National Weather Service Climate Prediction Center, http://www.cpc.noaa.gov).

**Fig. 13:**
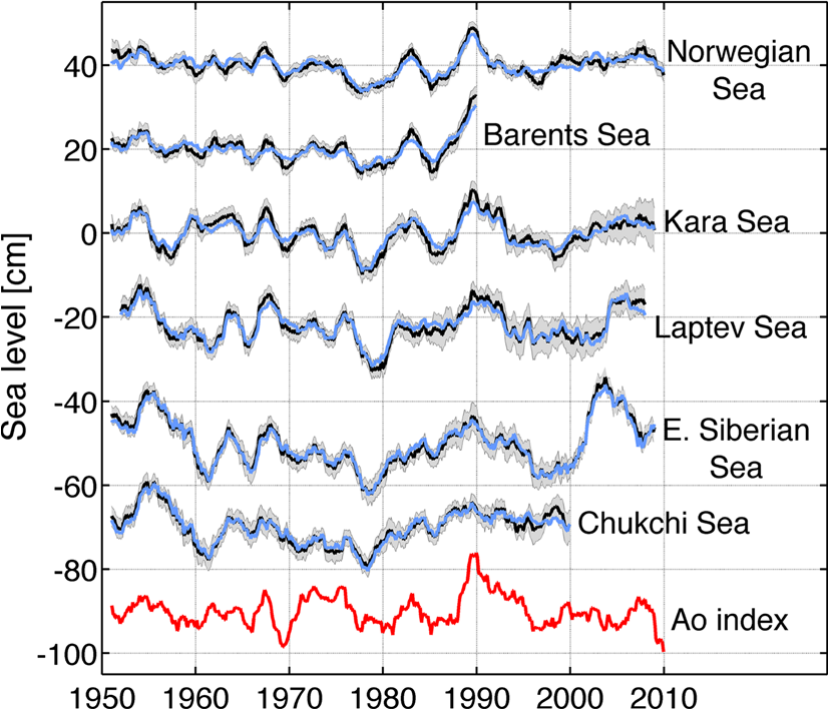
Low-pass-filtered (2-year running mean) time series of total (*black line*) and IB-corrected (*blue line*) sea level in six regions along the continental boundaries of the Arctic Ocean: in a counterclockwise order they are the Norwegian coast, Barents Sea, Kara Sea, Laptev Sea, East Siberian Sea, and Chukchi Sea. The AO index (*red line*) is also shown (scaled to have the same standard deviation as the average of the sea level time series). The *gray-shaded area* represents the uncertainty of the time series of total sea level. Adapted from Calafat et al. (2013)

In the past decade, several studies have focused on exploring the interannual-to-decadal sea level variability near the Norwegian coast and over the Arctic Ocean using tide gauge data, satellite observations and numerical models (e.g., Proshutinsky et al. 2001, 2004, 2007; Henry et al. 2012; Richter et al. 2012a, b; Volkov and Pujol 2012; Calafat et al. 2013). They found that both the IB effect and wind forcing are important in causing sea level variability, and the relative importance of the two has regional variations (e.g., Proshutinsky et al. 2001, 2004, 2007). They also found significant correlations between the AO index (red curve of Fig. 13) and 2- or 5-year running mean tide gauge records (black and blue curves of Fig. 13) but with distinct regional variations (Proshutinsky et al. 2007; Henry et al. 2012; Calafat et al. 2013). The correlations between AO index and regional mean sea level variability with IB effect (black curve) and without IB effect (blue curve) are 0.60 (0.56) for the Norwegian Sea, 0.79 (0.66) for the Barents Sea, 0.60 (0.46) for the Kara Sea, and 0.46 (0.37) for the Laptev Sea with all correlations exceeding the 95 % significance level. These correlations are based on the longest record available at each tide gauge station, with the longest record length being 1948–2010 (Table 1 of Calafat et al. 2013). Evidently, the AO can affect interannual and decadal sea level via the IB effect, as seen from the higher correlations with the IB influence (see above correlations outside the parentheses). The IB effect, however, may not be the dominant factor, because the correlations between the AO and sea level variability do not have dramatic decreases excluding the IB influence (correlations inside the parentheses), indicating that other factors (e.g., winds) associated with the AO are important in affecting Arctic sea level. In the East Siberian Sea and Chukchi Sea, the correlations are below 0.3 and not statistically significant above 95 % level.

The AO effects exhibit significant decadal changes, with stronger impacts (higher correlations) on sea level from 1950 to 1995 and almost vanishing correlations after 1995 in all regions (Poshutinsky et al. 2007; Henry et al. 2012; Calafat et al. 2013). Near the Norwegian coast, in the Barents Sea and Kara Sea regions where AO influences are stronger, local wind forcing and sea level signals propagating from the eastern boundary of the North Atlantic are the primarily causes for sea level variations (Richter et al. 2012b; Calafat et al. 2013). In the East Siberian, Chukchi, and also Laptev Seas where the AO is not a good indicator of sea level variations throughout the 1950–2010 period, sea level variability is very sensitive to the change of regional wind and SLP patterns, particularly the longshore wind, which can reproduce the major features of the observed sea level variability from 1950 to 1999. After 1999, sea level variability in these regions, especially in the East Siberian Sea, is associated with both the longshore wind and strengthening Beaufort Gyre (Calafat et al. 2013). A detailed study that quantifies the basin-wide spatial patterns of decadal sea level variability associated with the AO and the related governing processes in the Arctic Ocean is needed, including the local forcing over the Arctic and remote forcing from the Atlantic Ocean.

### The Southern Ocean

The Southern Annular Mode (SAM) is also referred to as the Antarctic Oscillation (Gong and Wang 1999; Limpasuvan and Hartmann 1999). As its northern hemisphere counterpart, the SAM is characterized by a deep, zonally symmetric structure with geopotential height anomalies of opposing signs in the polar cap region and in the surrounding zonal ring centered near 45°S (e.g., Thompson and Wallace 2000). The station-based SAM index is the pressure difference between the latitudes of 40°S and 65°S (Marshall 2003; See National Center for Atmospheric Research Climate Data Guide, https://climatedataguide.ucar.edu/, for SAM index since 1957). As such, the SAM index measures a “see-saw” of atmospheric mass between the middle and high latitudes of the Southern Hemisphere. Positive SAM index corresponds with stronger-than-average westerlies over the mid-high latitudes (50°S–70°S) and weaker westerlies in the midlatitudes (30°S–50°S).

Frankcombe et al. (2015) performed a multiple linear regression analysis by regressing the station-based SAM index onto 50-year SODA sea level and surface winds over the Indian and Pacific Oceans from 1958 to 2007 (bottom panels of Fig. 3). Their results suggest that the SAM is associated with significant sea level variability in the Southern Ocean including the southwest and southeast coasts of Australia, with visible signals in the tropical Pacific and Indian Oceans, but its influence is weak compared to the PDO, ENSO and IOD (Fig. 3). Similar conclusions can be drawn from satellite SSH data for the 1993–2007 period (Figure 3 of Frankcombe et al. 2015), even though this short period may alias the long-term trend with variability. The authors argued that the sea level signature in the equatorial Pacific associated with the SAM may partly reflect ENSO influence on the SAM, as suggested by existing studies (e.g., Ding et al. 2012).

Roemmich et al. (2007) suggested that the decadal spin up of the Subtropical Gyre over the South Pacific, which is associated with a 12-cm increase in satellite-observed SSH between 1993 and 2004 on large spatial scales centered at about (40°S, 170°W), results from the increased surface wind stress curl associated with an enhanced SAM. Regression analyses using a longer record of 1958–2007, however, do not show a strong influence of SAM in the South Pacific Subtropical Gyre region (Fig. 3, bottom row), suggesting that the influence of the SAM may be subject to significant decadal variability. On the other hand, the SAM can have apparent impacts on sea level and surface winds over the southern Indian Ocean (south of 30°S) and around Australia (bottom row of Fig. 3).

In addition to the zonally symmetric circumpolar forcing associated with SAM, there is evidence that zonal asymmetry in SAM may impact interbasin patterns of sea level change in the Southern Hemisphere. Thompson and Merrifield (2014) showed that a proxy for zonal differences in westerly wind strength over the Southern Ocean derived from SLP covaries with out-of-phase multidecadal sea level variations between the Indian-South Pacific and South Atlantic basins during most of the twentieth century. The authors hypothesized that this relationship is due to convergent and divergent transport in the Antarctic Circumpolar Current (ACC) forced by the zonal asymmetry in westerly wind strength. Zonal differences in recent westerly wind trends over the Southern Ocean are related to zonal differences in ocean bottom pressure trends in the ACC (Makowski et al. 2015).

## Summary, Issues and Challenges

This paper reviews the state of our knowledge about spatial patterns of sea level variability and underlying mechanisms for each ocean basin. It focuses particularly on the patterns associated with natural internal climate modes on decadal-to-multidecadal timescales. Over the Pacific Ocean, the PDO (IPO or decadal variability of ENSO) and NPGO are the two dominant climate modes and they are associated with distinct spatial patterns of sea level change, with both having global signatures (Sect. 2; Figs. 1, 2, 3, 5). Decadal change in ENSO behavior, however, can significantly alter its associated sea level patterns (Fig. 4). Winds associated with these modes (Fig. 6) are the primary causes for the overall sea level patterns, which are dominated by the thermosteric component, with the halosteric component often compensating the thermosteric sea level due to heat and salt redistribution by advection. Heat and freshwater fluxes, however, may also have significant contributions in some regions during some decades. Further studies are needed to quantify the effects of heat versus freshwater fluxes associated with the climate modes. Over the Indian Ocean, sea level trend patterns since the 1960s are driven primarily by surface winds (Fig. 7; Sect. 3), but the nature of the climate modes that contribute to the changing winds remains elusive. On decadal timescales, wind stress associated with ocean–atmosphere coupled modes—ENSO (which causes DIOB before the mid-1980s) and IOD (Fig. 8)—is the major cause for decadal sea level variability north of 20°S. The sea level pattern associated with the IOD alone shows an east–west dipole (Fig. 9), and frequently occurring intensified positive IOD in a warming climate could induce frequent SLRs in the western basin but falls in the eastern Indian Ocean during boreal fall.

Over the Atlantic, the NAO-associated sea level patterns exhibit a dipole structure in the North Atlantic basin (Figs. 10, 11). In the basin interior, surface heat fluxes are suggested to be the major force for the decadal sea level patterns due to AMOC variations (Sect. 4). Effects of freshwater flux and sea ice transport associated with the NAO remain to be explored. Along the eastern boundary, longshore winds and coastal Kelvin waves are the primary causes for the coherent sea level changes. Along the west boundary (US east coast), some studies demonstrate the importance of interior wind stress curl and local wind over the shelf in driving decadal sea level variability, whereas others argue for the importance of AMOC and Gulf Stream variations. The 20–30-year decadal sea level signals observed in both the North and South Atlantic (Fig. 12) are associated with AMOC variations and oceanic Rossby waves. The upward trend of the AMO SST index during recent decades coincides with the observed accelerated SLR along the US northeast coast. The variation of the AMO index, however, might be partly externally forced and thus may not represent the true internal AMO. Over the Arctic, significant correlations between the AO index and tide gauge records have been found, but with distinct regional variations (Fig. 13; Sect. 5). The correlation coefficients are high in the Barents Sea, Norwegian Sea and Kara Sea, weaker for the Laptev Sea and not significant for the East Siberian Sea and the Chukchi Sea. Winds and to a lesser degree IB effects are important for driving the sea level variability, and in the Norwegian Sea coastal signals propagating from the eastern boundary of the North Atlantic also contribute. Finally, the SAM can have a significant influence on sea level in the Southern Indian and Pacific Oceans, with weak influence over the tropics compared to the PDO, ENSO and IOD. Zonal asymmetry in SAM-associated winds might have contributed to the asymmetry of decadal sea level variations in the southern ocean during most of the twentieth century.

While progress has been made toward understanding the spatial patterns of sea level variability associated with internal climate modes, immediate issues and challenges for advancing our understanding remain. These issues must be resolved in order to achieve more accurate depictions of climate modes’ impacts on regional SLR and therefore contribute to improved decadal predictions and future projections of regional SLR. Relevant issues, challenges and future outlooks are discussed in each of the following aspects below. Understanding the impact of natural climate variability (coupled, forced or free internal ocean) on sea level is a prerequisite before anthropogenic finger prints can be identified.

*(a) Representation of climate modes and associated fields* Existing studies often use empirical analysis—particularly EOF—to identify climate modes. For instance, the PDO is defined as the leading EOF mode of SST in the North Pacific and its temporal variability is represented by PC1 (Sect. 2.1). While it is the leading empirical mode, the PDO is not a single physical mode of climate variability because it results from the combination of three groups of processes: (1) variability associated with the Aleutian low due to stochastic whether noise and remote forcing from the tropics (largely ENSO), (2) ocean memory and (3) decadal variability of Kuroshio–Oyashio currents induced by westward-propagating oceanic Rossby waves (see review paper by Newman et al. 2016). Given that different processes may dominate the PDO during different decades, the PDO-associated SST, wind, and thus sea level patterns may have different manifestations. Using a fixed SST pattern (EOF1) to represent the PDO may not depict these pattern changes, resulting in limitations for assessing its associated wind and sea level patterns. Improved PDO indices that are able to represent this temporal evolution need to be sought.

To extract the surface winds/sea level associated with climate modes, existing studies generally use multiple linear regression by regressing the observed or reanalysis winds/sea level onto climate mode indices. Note that the regression coefficients remain constant (static) in time, which measure their overall correlations for the examined temporal period. In reality, however, their correlations may exhibit strong decadal variability within the period of interest, due to behavior changes of climate modes; but the static linear regression method cannot depict this decadal variability. For instance, Andres et al. (2013) showed that the NAO is strongly correlated with interannual sea level variability along the US northeast coast since 1987, but they are almost uncorrelated before 1987 (from 1970 to 1987). Advanced statistical techniques that can extract the time-evolving surface wind/sea level patterns associated with climate modes will be very helpful.

*(b) Interactions among climate modes and effects of anthropogenic forcing* The climate modes over each ocean basin may not be fully independent. For example, the PDO and DIOB are highly correlated with positive correlation before the mid-1980s and negative correlation afterward (Fig. 8b); the PDO can affect Indian Ocean winds partly through modulating the Asian-Australian monsoon (Meehl and Arblaster 2011, 2012); decadal variability of tropical Indian Ocean SST since the mid-1980s and multidecadal trend since the 1960s (e.g., Han et al. 2014a) as well as tropical Atlantic warming since the 1990s (e.g., McGregor et al. 2014) might have affected the tropical Pacific winds and sea level; and changes in ENSO-PDO phase relationship may enhance decadal sea level variability in the western tropical Pacific (Moon et al. 2015).

Furthermore, SST indices of decadal climate modes (e.g., PDO and AMO) can be affected by anthropogenic warming and other external forcing (e.g., Dong et al. 2014; Booth et al. 2012; Knudsen et al. 2014). Consequently, winds and sea level patterns regressed onto these indices may not represent the effects of pure natural, internal modes. For these reasons, separating the effects of internal climate modes on sea level from that of external forcing (both anthropogenic and natural) using observational analysis remains a challenge. To this end, large ensemble experiments of climate models with long integrations using anthropogenic, natural, and all forcings, respectively, will be helpful for the separation, even though different climate models may suffer from different biases. Efforts are being made in this line. For example, the National Center for Atmospheric Research (NCAR) has completed a 42-member ensemble experiment using the Community Earth System Model (CESM) from 1920 to 2100, with historical all forcing from 1920 to 2005 and RCP8.5 trajectory since 2006 (Kay et al. 2015). The 42-member ensemble mean will damp internal climate modes and thus measure the effect of external forcing.

*(c) Effect of oceanic internal variability* Several recent studies examined the effects of oceanic internal variability (instabilities) on sea level variability, by performing experiments using standalone, eddy-permitting (1/3° × 1/3° and 1/4° × 1/4° grids) (e.g., Trenary and Han 2013; Li and Han 2015; Penduff et al. 2011) and eddy-resolving (1/12° × 1/12°) (Sérazin et al. 2015) OGCMs. Over the Indian Ocean, Trenary and Han (2013) and Li and Han (2015) suggested that oceanic internal variability has a significant contribution to decadal sea level variability in the subtropical south Indian Ocean, near the Somali coast and western Bay of Bengal. Penduff et al. (2011) demonstrated a significant increase of interannual SSHa variance by increasing OGCM resolution from 2° to ¼°. Sérazin et al. (2015) showed that the small-scale (*L* < 6°) SSHa variance is almost entirely of intrinsic origin at all timescales; the large-scale (*L* > 12°) low-frequency variability (*T* > 18 months), however, results largely from atmospheric forcing over most of the global ocean, but oceanic internal variability still has large amplitudes over the Gulf Stream, Kuroshio, and Antarctic Circumpolar Current regions. The intrinsic SSHa, which is independent of climate modes and unpredictable, affects the decadal predictability for regional sea level variability. Further research is required using high-resolution models to quantify the effects of oceanic intrinsic variability on decadal sea level variability and predictability.

*(d) Limited observational records* The short records of available datasets limit our ability to detect the full character of decadal climate variability. Modeling studies suggest that we need ~500 years of observations to sample the full range of ENSO decadal variability (e.g., Wittenberg 2009). Our reliable global-scale SST datasets based on in situ observations, however, are only ~one century long, and the records of in situ observed surface winds and sea level are even shorter. Yet, surface winds associated with climate modes are the major driver for the spatial patterns of sea level variability. Satellite observations (e.g., winds, sea level) have revolutionized our understanding of intraseasonal-to-interannual variability, but their records are too short for studying decadal variability. Therefore, these observations must be continued into the future to lengthen the record for studies of decadal climate variability.

Efforts have been made to generate reliable longer data records for climate studies (e.g., temperature, winds, sea level) by quality control existing historic datasets, assimilating observations into numerical models (reanalysis products), and applying statistical techniques to reconstruct basin- and global-scale datasets during earlier periods when observations are sparse in space. Apparent differences, however, exist among different reanalysis products, particularly with regard to their multidecadal trends (e.g., winds, Sect. 2.3). There are also significant differences among reconstructed sea level datasets before the satellite era regarding both global mean SLR (e.g., Christiansen et al. 2010; Calafat et al. 2014) and regional multidecadal trends over the Pacific (e.g., Moon et al. 2013). The discrepancies among different reconstructions are due primarily to different statistical approaches used before the satellite era (e.g., Christiansen et al. 2010; Calafat et al. 2014).

While significant progress has been made in producing various climate datasets, continuous effort is needed to further improve the quality of existing historical, reanalysis and reconstructed datasets, either by advancing our existing tools or by seeking new statistical techniques to overcome the known limitations. For documenting and understanding decadal scale variability, our existing satellite and in situ data records are too short and there remains an imperative for sustained in situ and satellite observations into the future. Since sea level represents the vertically integrated effects of ocean heat and freshwater uptakes, changes in surface and subsurface ocean circulations as well as their associated redistributions of heat, freshwater and mass, continued observations at the surface and subsurface are needed in order to understand the causes for decadal sea level variability, including those induced by climate modes. Recent advances in global observing system development—such as the Argo network that provides vertical temperature and salinity profiles in the upper 2000 m and satellite missions that provide accurate measurements of sea level (e.g., Jason series), ocean vector winds (e.g., international scatterometer missions) and sea ice extent/concentration—have substantially enhanced our capability to conduct sea level and climate research. These satellite and in situ measurements systems combine to provide indispensible observational resources for studying regional decadal variability. Sustaining and enhancing these observing systems, including enhancing deep ocean observations to measure hydrographic profiles below 2000 m, to obtain reliable and continuous data records are essential to ensure our future progress and success in understanding and predicting decadal sea level variability on regional scales.
